# Antibacterial Activity of Silver Nanoparticles (AgNP) Confined to Mesostructured, Silica-Based Calcium Phosphate against Methicillin-Resistant *Staphylococcus aureus* (MRSA)

**DOI:** 10.3390/nano10071264

**Published:** 2020-06-28

**Authors:** Jung-Chang Kung, Wei-Hsun Wang, Chung-Lin Lee, Hao-Che Hsieh, Chi-Jen Shih

**Affiliations:** 1 School of Dentistry, College of Dental Medicine, Kaohsiung Medical University, Kaohsiung 807, Taiwan; kung1129@kmu.edu.tw; 2 Department of Dentistry, Division of Family Dentistry, Kaohsiung Medical University Hospital, Kaohsiung 807, Taiwan; 3 Drug Development and Value Creation Research Center, Kaohsiung Medical University, Kaohsiung 807, Taiwan; 4 Department of Orthopedic Surgery, Changhua Christian Hospital, Changhua 500, Taiwan; wangweihsun@gmail.com; 5 School of Medicine, Kaohsiung Medical University, Kaohsiung 807, Taiwan; 6 Department of Medical Imaging and Radiology, Shu-Zen Junior College of Medicine and Management, Kaohsiung 821, Taiwan; 7 Department of Golden-Ager Industry Management, Chaoyang University of Technology, Taichung 413, Taiwan; 8 Department of Fragrance and Cosmetic Science, College of Pharmacy, Kaohsiung Medical University, Kaohsiung 807, Taiwan; dmon811317@gmail.com (C.-L.L.); jackie20633@hotmail.com (H.-C.H.); 9 Department of Medical Research, Kaohsiung Medical University Hospital, Kaohsiung 807, Taiwan

**Keywords:** antibacterial, AgNP, mesostructured silica, minimum inhibitory concentration (MIC)

## Abstract

*Staphylococcus aureus*, which is commonly found in hospitals, has become a major problem in infection control. In this study, Ag/80S bioactive ceramics used for enhanced antibacterial applications have been developed. An in vitro bioactivity test of the Ag/80S bioactive ceramic powders was performed in a phosphate-buffered saline (PBS). To explore the antibacterial activity of the Ag/80S bioactive ceramic powders, the Kirby-Bauer susceptibility test, the kinetics of microbial growth analysis and the colony-forming capacity assay were used to determine their minimum inhibitory concentration (MIC) against methicillin-resistant *Staphylococcus aureus* (MRSA). The results confirmed that the Ag/80S bioactive ceramic powders have antibacterial activity against MRSA (ATCC 33592) and MRSA (ATCC 49476).

## 1. Introduction

The World Health Organization has pointed out that the international public health issues of the new century are infectious diseases, and they will become one of the most serious diseases that threaten human health [1]. With the changes in human lifestyles, viruses or pathogenic microorganisms continue to adapt and mutate, leading to the emergence of various infectious diseases.

*Staphylococcus aureus* is a gram-positive bacterium that is naturally found on humans. In some cases, for reasons that are not currently known, it becomes a major problem in infection control. Because of the convenience of hospitalization and the country’s dense population, patients with *Staphylococcus aureus* infection are chronically ill or have been recently treated for different reasons [2]. Clinically, it is impossible to distinguish between nosocomial infections and community-type infections. In order to alleviate this problem, clinicians, scholars and experts have conducted research on the molecular resistance mechanisms of *Staphylococcus aureus*, which in recent years has become moderately resistant against vancomycin [3,4,5].

The osteomyelitis is caused by several bacteria interacting and 80% of this population of bacteria are *Staphylococcus aureus*. The generation factors of pathogenic bacteria can be divided into two categories: extrinsic factors (including trauma, surgery, bone traction and bone metal fixtures) and intrinsic factors (including systemic infections, diabetes, physical weakness, malnutrition and immunodeficiency). The current surgical treatments of osteomyelitis are antibiotics mixed with bone cement that are implanted into the infected part of the bone marrow. The treatment procedures include drainage, debridement (dead bone resection), antibiotic medicinal tamponade and flap transplantation. However, the limited variety of drugs available for treating antimicrobial-resistant pathogens may result in treatment failure or screening to more resistant strains. Pharmaceutical companies have turned to the research and development of antibacterial materials. Ag is one of the main candidates [6].

Ag played a bactericidal role before antibiotics were invented. It has a broad spectrum of antibacterial properties against Gram-positive and Gram-negative bacteria. Some bacteria are resistant to antibiotics, but Ag does not have this disadvantage [7,8,9,10,11,12,13]. Silver nanoparticles (AgNP) have an ability to inhibit both bacteria and viruses due to their strong reactivity. They have antibacterial properties at low concentrations [14,15,16,17] and their antibacterial ability is related to particle sizes. However, when they are used directly in clinical treatment, the following problems must be solved: whether Ag ions will reduce after their release and cause tissue staining, whether a large amount of Ag ions or AgNP will cause sudden release then damage to the tissue cells and whether it is toxic to human keratinocytes and fibroblasts [18,19]. Using bioceramics as an Ag carrier can be a solution.

For the first time, Ballontone et al. synthesized a silica-based calcium phosphate containing 3 wt.% Ag using a sol-gel method and found it was antibacterial against *Escherichia coli* (*E. coli*, MG1655) [20]. They further found that this material had antibacterial properties against *Staphylococcus aureus* (*S. aureus*, NCIMB 11852) and *Pseudomonas aeruginosa* (*PAO*, 6049) [21]. Up to now, the Ag-contained silica-based calcium phosphate has been successfully synthesized, and it can release Ag ions in suspension to achieve an antibacterial effect. However, the technical bottleneck of this field is that it can only inhibit general nonresistant bacteria. In contrast, due to its high specific surface area and mesoporous (2–50 nm) structures as well as the fact that the pore interface properties can be modified, mesostructured, silica-based calcium phosphate is more advantageous as a carrier for AgNP. Gargiulo et al. [22] and Phetnin [23] published and discussed the antibacterial activity of Ag-contained, mesostructured, silica-based calcium phosphate. However, they only discussed the common strains *S. aureus* (ATCC 6538, ATCC 25923) or *E. coli* (ATCC 25922).

This study mainly researches and synthesizes mesostructured, Ag-contained, silica-based calcium phosphate (80SiO_2_-CaO-P_2_O_5_+Ag bioactive ceramic powders referred to herein as Ag/80S). The developed Ag/80S has bioactivity and inhibits drug-resistant bacteria properties that can be used as an auxiliary material, and provides bone graft materials to have additional properties of osteoinductive, osteoconductive, and antibacterial.

In addition to explore the in vitro bioactivity of Ag/80S, the Kirby-Bauer susceptibility test, the kinetics of microbial growth analysis and the colony-forming capacity assay were analyzed, which determined the minimum inhibitory concentration (MIC) of Ag/80S against MRSA. In addition to the antibacterial dynamics of Ag/80S, this study also discusses the relationship between Ag/80S antibacterial activity and composition as well as time and dosage.

## 2. Materials and Methods 

### 2.1. Materials Preparation

The Ag/80S bioactive ceramic powders in this study belong to the SiO_2_-CaO-P_2_O_5_-Ag system, with composition ratios of Si, Ca and P in 80, 15 and 5 mole%, and the additional addition of Ag X mole%, where X = 0, 1 or 10 and is referred to as 80S, Ag1/80S and Ag10/80S, respectively.

The Ag/80S bioactive ceramic powders were prepared using a co-template process of the sol-gel technique [24]. The co-template was formed by thermal reversible hydrocolloid, Pluronic F-127 and polyurethane foam. The method for manufacturing 80S, Ag1/80S and Ag10/80S comprised steps of providing and mixing raw materials or precursors in 60 g ethanol (C_2_H_5_OH, 99.5%, ECHO Ltd., Miaoli, Taiwan) including tetraethyl orthosilicate (TEOS, 98%, ACROS Organics, Loughborough, Leicestershire, England) 6.7 g, calcium nitrate tetrahydrate (Ca(NO_3_)_2_·4H_2_O, 98.5%, SHOWA Ltd., Okayama, Japan) 1.43 g, triethyl phosphate (TEP, 98%, FLUKA Corp., New York, NY, USA) 0.73 g, a metal material precursor of silver nitrate (AgNO_3_, 99.8%, SHOWA Ltd., Okayama, Japan) added as mole% and a template surfactant of Pluronic F-127 (P-123, BASF SE, Ludwigshafen, Rheinland-Pfalz, Germany) 7 g, forming a mesoporous structure to form a mixture. Then, 2 M HNO_3_ 1.5 g was added into the mixture to adjust pH values. The mixture was synthesized to form an initial gel by sol-gel technique for 24 h. A three-dimensional macroporous configuration template of polyurethane foam was immersed in the initial gel at least once for aging and then dried at 100 °C for 24 h. The three-dimensional macroporous configuration template and the template surfactant of forming the mesoporous structure were removed during a heat treatment to form 1g of bioactive ceramic powder. The conditions of the heat treatment were starting from room temperature rising to 600 °C at a heating rate of 10 °C/min, held at this temperature for at least 2 h.

### 2.2. Textural Characterization

An X-ray diffraction (XRD) analysis was used to characterize the phase compositions and the in vitro bioactivity test of the Ag1/80S and Ag10/80S bioactive ceramic powders. XRD analysis was performed using an X-ray diffractometer (XRD-6000, Shimadzu, Japan). The conditions of the X-ray diffractometer were Cu Kα radiation (λ = 1.542 Å), Ni filter, operating current of 20 mA, operating voltage of 30 kV and step size of 4 °/min. The specific surface areas of the Ag/80S bioactive ceramic powder samples were analyzed and calculated using the Barret-Joyner-Halenda (BJH) method and the Brunauer–Emmett–Teller (BET) equation that were built into a specific surface area and porosimetry analyzer (ASAP 2010, Micromeritics Corp., Norcross, GA, USA). The adsorbent was Nitrogen.

The morphologies of the Ag/80S bioactive ceramic powder samples were performed using a field emission scanning electron microscope (FESEM) (Hitachi S-3000N, Hitachi, Tokyo, Japan).

### 2.3. In Vitro Bioactivity

The in vitro bioactivity tests were performed in phosphate-buffered saline (PBS, Cat. No. CC702-0500, GeneDireX, Inc., Taoyuan, Taiwan). The composition and ionic concentration of the PBS were similar to those of human plasma. The Ag/80S samples were immersed in PBS to test the in vitro bioactivity with a solid to liquid ratio of 1 mg/mL at 37 °C to monitor the formation on the Ag/80S samples surface after 0 h, 24 h, 48 h, 72h and 168h, respectively. SEM techniques were used to analyze the in vitro bioactivity of the Ag/80S samples.

### 2.4. Ag/80S Bioactive Ceramic Powders Characterization

The Ag/80S powders at concentrations of 5 mg/mL~20 mg/mL were added in a tryptone soy broth (TSB, Neogen^®^Corporation, Lansing, MI, USA) to form a mixture. Then, the mixture was oscillated at 160 rpm for 24 h, and the Ag/80S particles were removed with a 0.22-μm filter to obtain a colloid (referred to herein as Ag/80S). UV absorption spectra of the Ag/80S were taken on a UV/Vis spectrophotometer (Beckman Coulter, Inc., DU-800, Fullerton, CA, USA) with a range of 200 nm~600 nm. A variable path bucket with quartz windows was used. The size and the degree of dispersion of the Ag/80S were assessed by transmission electron microscopy (TEM, JEM-2100F, JEOL Ltd., Tokyo, Japan).

### 2.5. Bacterial Incubation and Antibacterial Activity

In this study, we selected S. aureus (ATCC 6538), MRSA (ATCC 33592) and MRSA (ATCC 49476) for the antibacterial activity tests.

#### 2.5.1. Disc Diffusion Method

Each 0.05 g of three different stoichiometric Ag/80S bioactive ceramic powders (80S, Ag1/80S and Ag10/80S) was pressed into a disc by a press of 2000 Pa and 30 s with a hydraulic lab press machine (MPT-63-100-10-3T, MARTO, Taiwan). After an autoclave sterilization procedure was completed, the disc was completely dried at 60 °C for 24 h. 10^8^ CFU/mL of *S. aureus* (ATCC 6538), MRSA (ATCC 33592) and MRSA (ATCC 49476) suspensions were respectively incubated in a tryptone soy broth (TSB, Neogen^®^Corporation, Lansing, MI, USA) at 37 °C for 24 h by an incubator (FORMA STERI-CYCLE CO_2_ Incubator, Thermo Fisher Scientific, Waltham, MA, USA). 200 μL of the suspensions was taken with a triangular glass rod to spread on a tryptone soy agar (TSA), respectively. The TSA was formed by dissolving 30 g of the tryptone soy broth (TSB, Neogen^®^Corporation, Lansing, MI, USA) powder and 15 g of the Agar powder (AG005, Bioresolustion, Taiwan) in 1 L of purified water, mixing thoroughly, then putting in the autoclave at 121 °C for 15 min. The sterilized discs were fixed on the TSA and were incubated at 37 °C for 24 h. The antibacterial activity of the discs were evaluated by observing the inhibition zones around the discs.

#### 2.5.2. Kinetics of Microbial Growth

The microbial growth kinetics of the Ag/80S against MRSA or S. aureus was carried out by microplate method. The minimum inhibitory concentration (MIC) is the lowest concentration of an antibacterial material to inhibit visible growth. The Ag/80S formed in the study was added or not added in an environment or system containing a liquid medium (such as TSB) and microorganisms. After being incubated at 37 °C for 24 h, each group of experiment suspension was formed. The number of microorganisms of each group of the experiment suspension was measured, wherein the turbidity of a certain amount of the experiment suspension was measured and converted by a turbidity meter (DEN-1, Biosan, Latvia) to obtain the number of microorganisms [25]. The turbidity of the experiment suspension was measured by an absorbance value (OD value) at a wavelength of 600 nm by a spectrophotometer (Infinite^®^ F50, TECAN, Switzerland). Taking time as the horizontal axis and absorbance (OD_600_) as the vertical axis, the growth curve of the experiment microorganisms in this environment or system was plotted. The condition of the liquid medium is that the ratio of the weight (mg) of the Ag1/80S extract to the volume (mL) of TSB, is 5, 10 and 20. The culture was incubated at 37 °C, 160 rpm for 24 h, and centrifuged at 3000 rpm for 5 min, the supernatant was then aspirated, which was the extract of the experiment sample. The experiment microorganisms were thawed and inoculated on each agar, and then incubated in each specific temperature incubator for a specific period of time. The strains were scraped with sterile cotton swabs and inoculated into each sterile liquid medium. The experiment bacterial suspension was measured by a turbidity meter, and its concentration was adjusted to about 1.5 × 10^8^ CFU/mL (colony-forming units per milliliter) [26]. The suspension was added to a 96-well microtiter plate in which the extract of the experiment sample was prepared, and the concentration of the bacterial suspension in each well was about 5 × 10^5^ CFU/mL after the inoculation. The bacterial suspension was incubated at 37 °C, and the absorbance was measured by a spectrometer once every hour to 24 h, and the kinetics curve of microbial growth was plotted to find the MIC of the Ag1/80S on different microorganisms. The MIC refers to the minimum concentration at which the growth of microorganisms can be inhibited and observed after 24 h of cultivation. The control group condition was a liquid medium containing each of the different experiment strains, incubated for 24 h without adding the respective experiment suspensions containing the Ag1/80S.

#### 2.5.3. Colony-Forming Capacity Assay

Since the kinetics curves of microbial growth plotted in this study can only show the growth curve changes of bacteria by numerical and computer drawing methods, it is impossible to observe the bacterial growth after Ag1/80S co-cultivation with bacteria (activities and strain morphology changes). In response to this situation, we further used a colony-forming capacity assay to analyze bacterial samples that had completed the kinetics of microbial growth. The principle is that after the bacteria are co-cultured with Ag1/80S, the colony-forming ability is changed due to the Ag1/80S. If the bacteria have been inhibited by Ag1/80S, the colony-forming ability will be reduced. Otherwise, if the antibacterial suspension has no inhibitory effect on the bacteria, the colony-forming ability will not be changed. This method can be used to double check the Ag1/80S of minimum bactericidal concentration (MBC) and minimum inhibitory concentration (MIC) against bacteria. The steps of a colony-forming capacity assay are to dip the bacteria-containing suspensions by sterilized cotton swabs after the kinetics of microbial growth analysis, and apply them to an agar which is divided into lines. The agar is incubated at 37 °C for 24 h, then photos are taken and observed. 

## 3. Results

### 3.1. Textural Characterization of the Ag/80S Bioactive Ceramic Powders

Table 1 shows the specific surface areas, mean pore sizes and pore volumes obtained from the desorption branch of the isotherms for the 80S, Ag1/80S and Ag10/80S bioactive ceramic samples. The mean mesopore size of all samples ranged from 6.2 to 7.5 nm. The surface areas of all samples ranged from 348.9 m^2^/g to 264.5 m^2^/g. Obviously, the surface area decreases as the proportion of added Ag increases. However, all of the Ag/80S samples have relatively high surface areas and porosity compared with conventional silver-incorporated bioactive ceramic powders [27].

### 3.2. Phase Compositions and In Vitro Bioactivity of Ag/80S Bioactive Ceramic Powders

**Figure 1:**
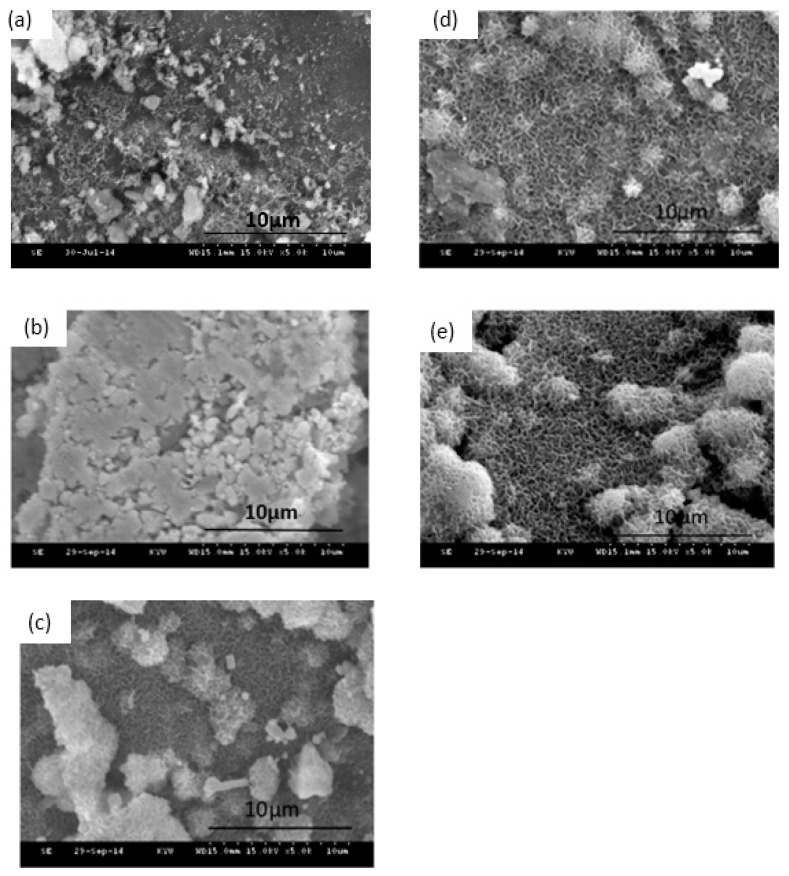
SEM images of Ag1/80S after immersion in phosphate-buffered saline (PBS) for different times: (**a**) 0 h; (**b**) 24 h; (**c**) 48 h; (**d**) 72 h; and (**e**) 168 h.

**Figure 2:**
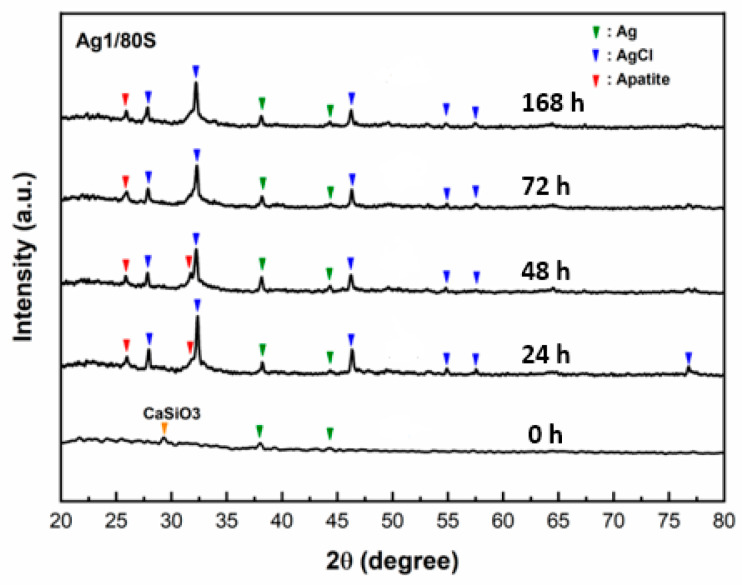
XRD patterns of Ag1/80S after immersion in PBS for different times.

An important property of bioactive materials is their ability to bond to living tissue, such as bone tissue. The formation of an apatite-like layer on the surface of bioactive materials was observed to understand the occurrence of bonding to bone tissue in vitro and in vivo [28]. The SEM images of the Ag1/80S after being immersed in PBS for 0 h, 24 h, 48 h, 72 h and 168 h are shown in the Figure 1. These surfaces of Ag1/80S show significant changes after being immersed in PBS for different times. The Ag1/80S surface is smooth after being immersed in PBS for 24 h. The surfaces are all fully covered with a layer of needle-like crystallites for 48 h, 72 h and 168 h. Furthermore, the spherical particles with needle-like crystallites increase with increasing immersion time. These results indicate that these Ag1/80S can induce the formation of an apatite-like layer on their surfaces when immersed in PBS. The XRD patterns of the Ag1/80S before and after immersion in PBS are shown in Figure 2. Before immersing in PBS, the XRD patterns of Ag1/80S exhibit several diffraction peaks: one appeared at 29.3° and corresponds to a calcium-silicate-like phase according to JCPD 43-1460; others appeared at 38.1° and 44.3° and correspond to the (111) and (200) of the Ag according to JCPD 89-3722. After immersing in PBS for 24 h~168 h, XRD patterns of all Ag1/80S exhibit several new diffraction peaks, including 25.9° and 31.7°, which correspond to the (002) and (211) of the apatite-like phase according to JCPD 89-4405, 38.1° and 44.3°, which correspond to Ag according to JCPD 89-3722, and 27.8°, 32.2°, 46.2°, 54.8° and 57.5°, which correspond to the (111), (200), (220), (311) and (222) of the silver chloride (face-centered cubic phase) according to JCPD 85-1355. These are described as Ag1/80S and can induce the formation of an apatite-like layer on their surfaces after being immersed in PBS for more than 24 h.

**Figure 3:**
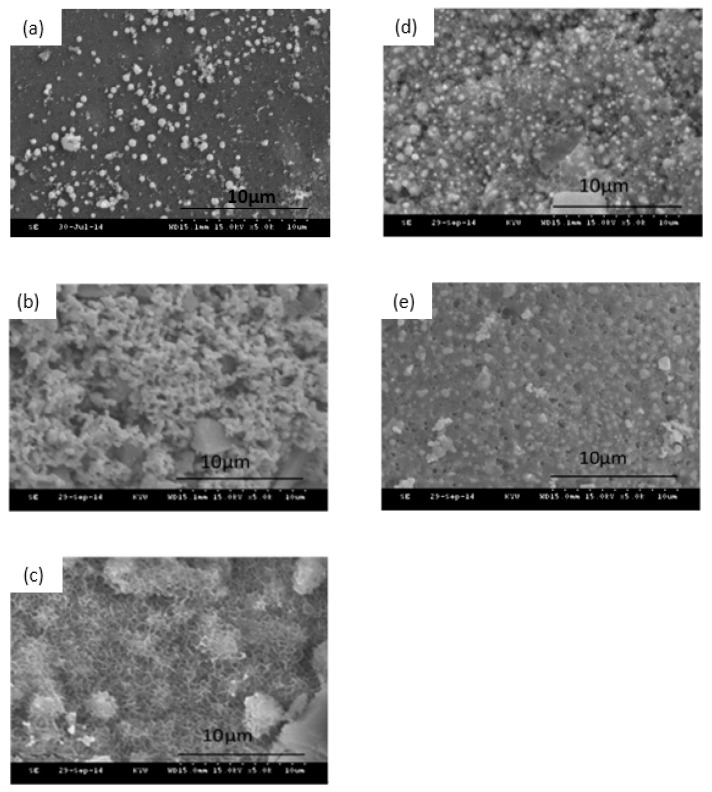
SEM images of Ag10/80S after immersion in PBS for different times: (**a**) 0 h; (**b**) 24 h; (**c**) 48 h; (**d**) 72 h; and (**e**) 168 h.

**Figure 4:**
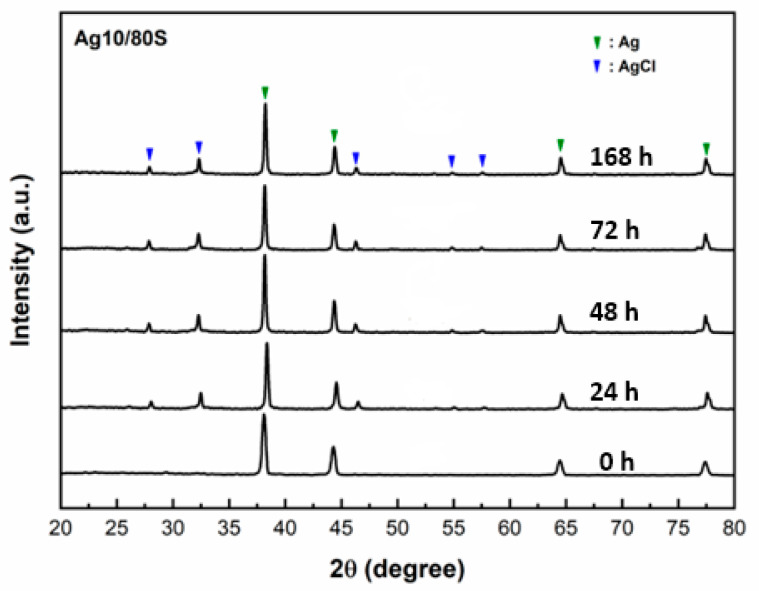
XRD patterns of Ag10/80S after immersion in PBS for different times.

The SEM images of the Ag10/80S after being immersed in PBS for 0 h, 24 h, 48 h, 72 h and 168 h are shown in Figure 3. Compared with different immersion times, only a few needle-like crystallites are observed on the surface of Ag10/80S after being immersed in PBS for 48 h. The XRD patterns of Ag10/80S verify this phenomenon as shown in Figure 4, and only the diffraction peaks of silver and silver chloride are present. A comparison of the SEM images and XRD patterns of Ag1/80S and Ag10/80S demonstrates that excessive silver addition apparently inhibits apatite-like growth.

### 3.3. Characterization of the Ag/80S Bioactive Ceramic Powders

**Figure 5:**
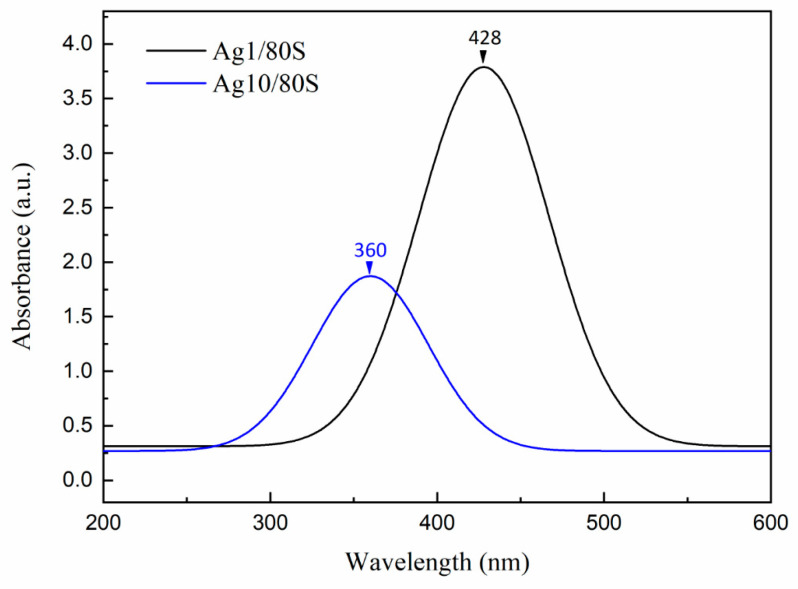
UV-Vis spectrums of Ag1/80S; and Ag10/80S.

**Figure 6:**
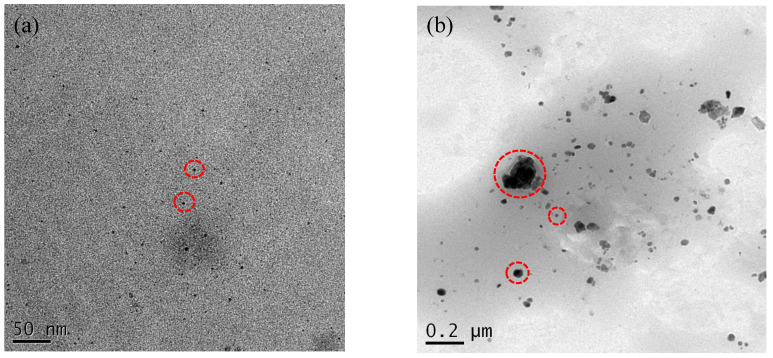
TEM images of (**a**) Ag1/80S; and (**b**) Ag10/80S (Within each red circle is a single AgNP).

The UV-vis spectra of Ag1/80S and Ag10/80S are shown in Figure 5. They show that the Ag1/80S has a maximum absorption peak at 428 nm, and the Ag10/80S has a maximum absorption peak at 360 nm, which confirmed the presence of AgNP. The TEM images of Ag1/80S and Ag10/80S are shown in Figure 6. They show two completely different morphologies. In the Ag1/80S samples, the AgNP have an average particle size of about 5 nm and a uniform particle size distribution. Nevertheless, in the Ag10/80S samples, the particle size range of the AgNP is from 5 nm to 200 nm, and the particle size distribution is not uniform.

### 3.4. Microbiological Assay of 80S and Ag/80S Bioactive Ceramic Powders

#### 3.4.1. Disc Diffusion Method Analysis

**Figure 7:**
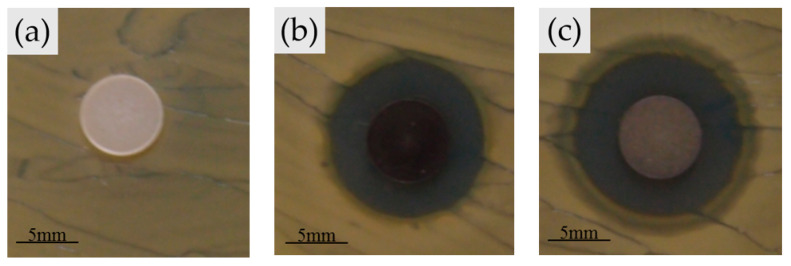
Disc diffusion method of (**a**) 80S; (**b**) Ag1/80S; and (**c**) Ag10/80S powder disc against *Staphylococcus aureus* (*S. aureus*, ATCC 6538).

Figure 7 shows that the differences in the inhibition zone size around all of the powder discs (80S, Ag1/80S and Ag10/80S) against *S. aureus* (ATCC 6538) are significant. No inhibition zone formed around the powder disc of 80S. The 80S powder disc has no antibacterial effect because it does not contain silver. The Ag1/80S powder disc and the Ag10/80S powder disc have antibacterial effects against *S. aureus* (ATCC 6538). The diameters of the inhibition zones formed around the powder discs are ranged from 16 mm to 19 mm. The antibacterial effect of Ag1/80S and Ag10/80S are attributed to the release of Ag^+^ from the powder discs, which indicate that the increase in Ag^+^ release resulted in the increased antibacterial activity of *S. aureus* (ATCC 6538).

#### 3.4.2. Kinetics of Microbial Growth Analysis and Colony-Forming Capacity Assay Analysis

The results of the disc diffusion method analysis show that the antibacterial effect is observed due to the occurrence of an inhibition zone around Ag1/80S. To explore the detailed process, we used the kinetics of microbial growth analysis to investigate the correlation between colony number (OD_600_) and time. 

**Figure 8:**
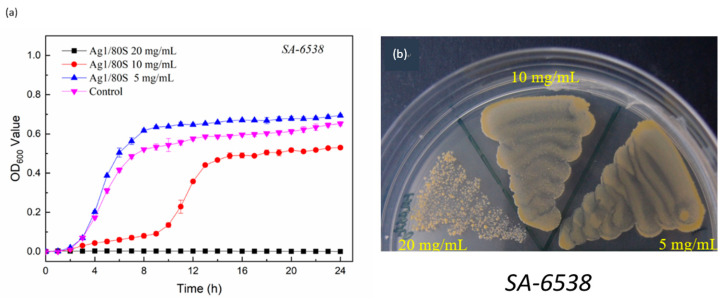
Microbiological assay of Ag1/80S against *S. aureus* (ATCC 6538); (**a**) kinetics curves of microbial growth; and (**b**) colony-forming capacity assay.

The results corresponding to the correlation between colony number (OD_600_) and time at different Ag1/80S concentrations are shown in Figure 8a. When the Ag1/80S concentration is 5 mg/mL, the growth curve of *S. aureus* (ATCC 6538) is similar to that of the control group. The kinetics curve of microbial growth is divided into two stages. Stage 1 is before hour 8. *S. aureus* (ATCC 6538) is in a growth period, and the absorbance values (OD_600_) are rapidly increased due to its rapid propagation. Stage 2 is after hour 8. *S. aureus* (ATCC 6538) is in a gentle declining period, and the absorbance values (OD_600_) are slightly increased due to its growth saturation. This result indicates that 5 mg/mL of Ag1/80S cannot inhibit the growth of *S. aureus* (ATCC 6538). 

When the Ag1/80S concentration is 10 mg/mL, the kinetics curve of microbial growth is divided into three stages. Stage 1 is before hour 10 and can effectively inhibit the growth of *S. aureus* (ATCC 6538) in this period. Stage 2 is from hours 10 to 13, during which the absorbance values (OD_600_) are increased rapidly due to rapid propagation of *S. aureus* (ATCC 6538) in this period. Stage 3 is from hours 13 to 24, during which *S. aureus* (ATCC 6538) becomes saturated during a gentle declining period, which results in a slight increase of OD_600_. This result indicates that 10 mg/mL of the Ag1/80S can inhibit the growth of *S. aureus* (ATCC 6538) during Stage 1. 

When the Ag1/80S concentration is 20 mg/mL, the absorbance values (OD_600_) show a flat trend during the experiment interval, which indicates that 20 mg/mL of the Ag1/80S can effectively inhibit the growth of *S. aureus* (ATCC 6538).

The kinetics curves of microbial growth can only be resolved when the bacteria grow, because the curves cannot show the number of live bacteria and dead bacteria in a bacterial tube by the absorbance value (OD_600_). For clarity, the MIC values of the Ag1/80S against *S. aureus* (ATCC 6538) were determined using a colony-forming capacity assay, and the results are shown in Figure 8b. When the Ag1/80S concentration is 20 mg/mL, the colonies formed are separate. Although there are still colonies, the absorbance of the kinetics curve of microbial growth does not increase compared with hour 0, indicating that the Ag1/80S in this concentration has reached the MIC range. Therefore, it can be proved that an Ag1/80S concentration of 20 mg/mL can effectively inhibit the growth of *S. aureus* (ATCC 6538). 

**Figure 9:**
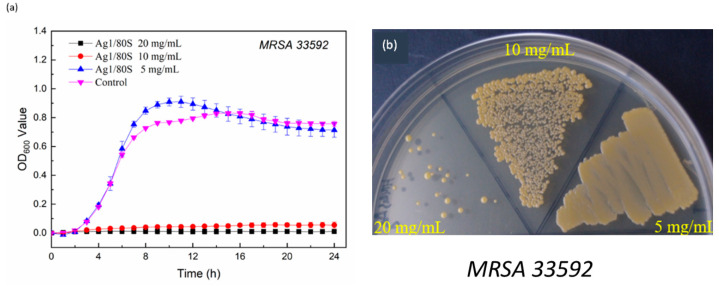
Microbiological assay of Ag1/80S against MRSA (ATCC 33592); (**a**) kinetics curves of microbial growth; and (**b**) colony-forming capacity assay.

**Figure 10:**
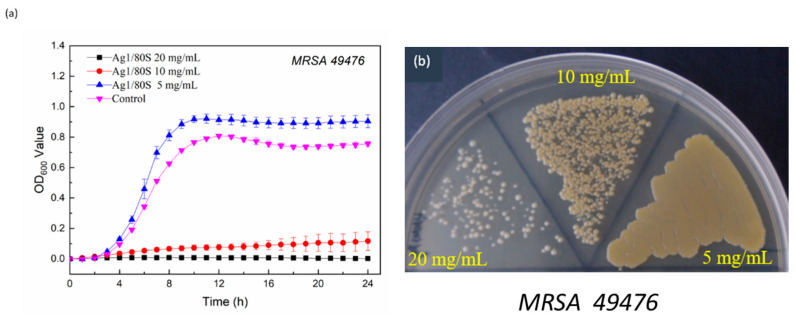
Microbiological assay of Ag1/80S against MRSA (ATCC 49476); (**a**) kinetics curves of microbial growth; and (**b**) colony-forming capacity assay.

Two common clinical strains of clinical hip infections, MRSA (ATCC 33592) and MRSA (ATCC 49476), were used to further verify the inhibition of the Ag1/80S against drug-resistant bacteria in this study. The results are shown in Figure 9a and Figure 10a. Similarly, when the concentration of the Ag1/80S is 5 mg/mL, MRSA (ATCC 33592) and MRSA (ATCC 49476) of the kinetics curves of microbial growth are similar to the control group. The kinetics curve of microbial growth is divided into two stages. Stage 1 is before hour 8 when MRSA (ATCC 33592) and MRSA (ATCC 49476) are in a growth period, and the absorbance values (OD_600_) are rapidly increased due to their rapid propagation. Stage 2 is after hour 8, during which MRSA (ATCC 33592) and MRSA (ATCC 49476) are in a gentle declining period, and the absorbance values (OD_600_) are slightly increased due to their growth saturation. When the Ag1/80S concentration is 10 mg/mL or 20 mg/mL, the absorbance values (OD_600_) show a flat trend during the experiment interval, which indicates that 10 mg/mL or 20 mg/mL of the Ag1/80S can effectively inhibit the growth of MRSA (ATCC 33592) and MRSA (ATCC 49476).

For clarity, the MIC of the Ag1/80S against MRSA (ATCC 33592) and MRSA (ATCC 49476) were analyzed and determined by a colony-forming capacity assay, and the results are shown in Figure 9b and Figure 10b. When the Ag1/80S concentration is 10 mg/mL, the colonies formed are separate, indicating that the Ag1/80S at this concentration limited the growth of the bacteria even if the minimum bactericidal concentration (MBC) is not reached. When the Ag1/80S concentration is 20 mg/mL, there are very few colonies, indicating that the Ag1/80S at this concentration has a higher growth limitation to the bacteria. Because there are some colonies, it is still within the range of MIC, and MBC is not reached. Therefore, it can be proved that 10 mg/mL and 20 mg/mL of the Ag1/80S can effectively inhibit the growth of MRSA (ATCC 33592) and MRSA (ATCC 49476). 

## 4. Discussion

Silica-based calcium phosphate (SiO_2_-CaO-P_2_O_5_) has the advantages of being osteoinductive and osteoconductive, having a low degradation rate and combining with soft tissue or hard tissue to help bone tissue regeneration, Therefore, it has been widely used to repair bone defects [22]. From Figure 1, Figure 2, Figure 3 and Figure 4 of this study results show that Ag1/80S can induce the formation of an apatite-like layer on their surfaces after being immersed in PBS for 48 h. The osteoinductive property of the Ag1/80S is better than that of the Ag10/80S. Research on silica-based calcium phosphate applied in antibacterial applications has been published [22,29]. It has an antibacterial ability due to the continuously released alkaline substances [21]. As the particle sizes decrease or the rate of release of alkaline substances increases, its antibacterial ability can be enhanced [30]. From Figure 6 of this study, results show that the particle sizes of the Ag1/80S are smaller than that of the Ag10/80S. Therefore the antibacterial ability of the Ag1/80S is better than that of the Ag10/80S. The future work of our team is to add Ag1/80S to commercial bone cement such as polymethyl methacrylate (PMMA). The antibacterial Ag1/80S will be used as auxiliary material for bone-grafting material, which can make the commercial bone cement (PMMA) have additional osteoinductive, osteoconductive and antibacterial properties. The development of bone-graft materials that can be applied to the human body is the ultimate goal of our team.

After immersing in PBS for 24 h~168 h, XRD patterns of all Ag1/80S exhibited the silver chloride phases in Figure 2 of this study. Gargiulo et al. [30] reported that the possible reason for the existence of the AgCl phase after immersing Ag-MBGs in PBS was the release of Ag ions that immediately reacted with the chlorides in the PBS. Therefore, the highly crystalline AgCl (insoluble salt) is found on the surface of the spherical particles with needle-like crystallites. The content of the metallic silver phase decreases after immersion in PBS due to the release of Ag ions and the well-crystalized AgCl formation.

A correlation between the particle size (d) and surface plasmon resonances (SPRs) has been reported [31]. A singular absorption band at around 420 nm is assigned to the surface plasmon resonance of Ag nanoparticles. Through the blue-shift and red-shift of the peak, the spectrum indicates Ag particles in different size. The SPR absorption band exhibits a special behavior: when it decreases in size from d ≈ 20 nm, it undergoes a blue-shift, then, when the size is near d ≈ 12 nm, it undergoes a strong red-shift. The multilayer Mie theory model agrees well with the observations, indicating that lowered electron conductivity in the outermost atomic layer due to chemical interactions is the cause of the red-shift. Based upon the UV-vis spectra of Figure 5 and TEM images of Figure 6 in this study, it is therefore believed that, regarding the size-dependence of surface plasmon resonances, the maximum absorption peak is from 360 nm (Ag10/80S) to 428 nm (Ag1/80S). The results are consistent with the AgNP of Ag1/80S which have an average particle size of about 5 nm; the particle size range of the AgNP in Ag10/80S are from 5 nm to 200 nm.

The Ag1/80S formed in this study can inhibit MRSA (ATCC 33592) and MRSA (ATCC 49476). We could speculate the reason of the antibacterial activity is that the Ag metal in the Ag1/80S is toxic to bacterial cells. The possible antibacterial mechanisms of silver nanoparticles can be found in recent papers [32,33]. In this study, the possible antibacterial mechanisms of Ag1/80S is small molecules (e.g., reactive oxygen species, ROS) in the Ag1/80S could attack the core of the Ag nanoparticles to induce more ROS. When Ag atoms reached a certain concentration, they have electron-sharing affinities that can result in formation of covalent bonds with S in protein, which can lead to the formation of protein dysfunction and the depletion of antioxidant. Aerobic respiration readily gives rise to H_2_O_2_ and O_2_^•−^, and they ultimately lead to bacteria death [34].

## 5. Conclusions

In this study, the in vitro bioactivity and antibacterial evaluation of Ag/80S bioactive ceramic powders against *S. aureus* and MRSA were studied. The results of this paper are now summarized:

1. Ag1/80S can induce the formation of an apatite-like layer on their surfaces after being immersed in PBS for 48 h. XRD patterns of Ag10/80S verify only the diffraction peaks of silver and silver chloride existence. Excessive silver addition apparently inhibits the apatite-like layer growth.

2. In the Ag1/80S samples, the AgNP have an average particle size of about 5 nm and a uniform particle size distribution. Nevertheless, in the Ag10/80S samples, the AgNP have particle sizes from 5 nm to 200 nm, and the particle-size distribution is not uniform. 

3. The results of the kinetics of microbial growth analysis and the colony-forming capacity assay confirmed that the Ag1/80S powders have antibacterial activity against *S. aureus* (ATCC 6538), MRSA (ATCC 33592) and MRSA (ATCC 49476).

4. The MIC of the Ag1/80S bioactive ceramic powders against *S. aureus* (ATCC 6538) can be lower than 20 mg/mL but for sure higher than 10 mg/mL, that against MRSA (ATCC 33592) can be a bit less than 10 mg/mL, but for sure not close to 5mg/mL, and that against MRSA (ATCC 49476) can be a little higher but close to 10 mg/mL. In no cases was the minimum bactericidal concentration (MBC) verified.

## Figures and Tables

**Table 1:** Brunauer–Emmett–Teller (BET) equation results of Ag/80S for different compositions.

	Surface Area (m^2^/g)	Pore Volume (cm^3^/g)	Mean Pore Size (nm)
80S	348.9	0.72	7.3
Ag1/80S	307.6	0.60	7.5
Ag10/80S	264.5	0.41	6.2
